# Gut microbial compositions mirror caste‐specific diets in a major lineage of social insects

**DOI:** 10.1111/1758-2229.12728

**Published:** 2019-01-08

**Authors:** Saria Otani, Mariya Zhukova, N'golo Abdoulaye Koné, Rafael Rodrigues da Costa, Aram Mikaelyan, Panagiotis Sapountzis, Michael Poulsen

**Affiliations:** ^1^ Section for Ecology and Evolution, Department of Biology, Universitetsparken 15 University of Copenhagen DK‐2100 Copenhagen Denmark; ^2^ Department of Natural Sciences (UFR‐SN) Nangui Abrogoua University 28 BP 847, Abidjan 28 Côte d'Ivoire; ^3^ Department of Entomology and Plant Pathology North Carolina State University Raleigh, North NC 27605 USA

## Abstract

Social insects owe their ecological success to the division of labour between castes, but associations between microbial community compositions and castes with different tasks and diets have not been extensively explored. Fungus‐growing termites associate with fungi to degrade plant material, complemented by diverse gut microbial communities. Here, we explore whether division of labour and accompanying dietary differences between fungus‐growing termite castes are linked to gut bacterial community structure. Using amplicon sequencing, we characterize community compositions in sterile (worker and soldier) and reproductive (queen and king) termites and combine this with gut enzyme activities and microscopy to hypothesise sterile caste‐specific microbiota roles. Gut bacterial communities are structured primarily according to termite caste and genus and, in contrast to the observed rich and diverse sterile caste microbiotas, royal pair guts are dominated by few bacterial taxa, potentially reflecting their specialized uniform diet and unique lifestyle.

## Introduction

Division of labour is a cornerstone in social insects (ants, termites and a number of wasps and bees) (Eggleton, 2011; Ferguson‐Gow *et al.,* 2014; Johnson, 2010; Li *et al.,* 2015) and associations with microbial symbionts have also played key roles in their success (Batra and Batra, 1979; Engel *et al.,* 2009; Lanan *et al.,* 2016; Sapountzis *et al.,* 2015). This applies also to termites, which evolved from cockroaches about 150 Mya, and where extensive work has shed light on the presence and role of gut bacterial communities (Brune, 2014; Brune and Dietrich, 2015; Douglas, 2015). Evolutionarily lower and higher termites have compositionally distinct gut microbiotas, shaped by diet and evolutionary histories with hosts (Dietrich *et al.,* 2014; Mikaelyan *et al.,* 2015a; 2017; Bourguignon *et al.,* 2018). The higher termites diversified their diets to include soil, humus, leaf litter, grass, dung and fungal material, coinciding with the loss of gut protists and the diversification of bacterial gut assemblies (Brune and Ohkuma, 2011; Brune, 2014; Brune and Dietrich, 2015; Rahman *et al.,* 2015; Mikaelyan *et al.,* 2015a; 2017).

Mature higher termite colonies produce reproductive alates that are believed to bring gut microbes from their natal nest as an inoculum for the first worker cohort of incipient nests (Brune and Dietrich, 2015; Rahman *et al.,* 2015; Benjamino and Graf, 2016). Within colonies, the queen and king reproduce, soldiers defend the colony and workers forage for food, maintain colony structures and care for the brood, soldiers and the royal pair (Eggleton, 2011). However, although it is well established that gut bacteria play essential roles in termites, our understanding of compositional and functional differences in bacterial communities across castes is limited (but see e.g. Benjamino and Graf, 2016; Berlanga *et al.,* 2011; Hongoh *et al.,* 2006; Li *et al.,* 2015, 2016; Poulsen *et al.,* 2014).

The higher termite subfamily Macrotermitinae cultivate the fungal symbiont *Termitomyces* (Sands, 1969; Aanen *et al.,* 2002) in fungus gardens (combs) to produce nutrient‐ and conidia‐rich nodules that are consumed by young workers, who redeposit conidia mixed with plant substrate as new comb after a first gut passage (Badertscher *et al.,* 1983). Older workers ingest mature fungus comb (Badertscher *et al.,* 1983; Leuthold *et al.,* 1989) and the soldiers, queen, and king are fed by workers (Hongoh *et al.,* 2006; Leuthold *et al.,* 2004). Division of labour and differences in diet among castes could be integrated with gut microbial compositions. For instance, *Macrotermes gilvus* worker and soldier gut communities are shaped more by termite age than caste (Hongoh *et al.,* 2006) and *Odontotermes formosanus* communities vary by gut compartments in workers of different ages (Li *et al.,* 2016).

No studies have carried out a comprehensive comparison of quantitative differences in microbiota composition between castes from different farming termite species within and between colonies sampled from geographically distant sites in nature. We characterized gut microbiotas using MiSeq amplicon sequencing on worker and soldier guts from colonies of three termite species spanning more than 350 km in South Africa. We supplement our community profiling with fluorescence *in situ* hybridisation (FISH), light and confocal microscopy and gut enzyme profiling to elucidate bacterial location and enzymatic differences across castes. Lastly, we comparatively analyse queen and king gut microbiotas from eight species of fungus‐growing termites from the Ivory Coast to explore whether the unique lifestyle of the royal pair shapes gut microbiota compositions.

## Results and discussion

### *Sequencing and bacterial identification*

Sequencing of bacterial communities of 180 sterile and reproductive termite caste gut samples of *Macrotermes natalensis*, *Odontotermes* cf. *badius* and *Odontotermes* sp. using Illumina MiSeq amplicon sequencing (Reagent Kit V2 500) resulted in 3 047 202 quality‐filtered sequences and 12 610 bacterial OTUs (Supporting Information Tables S1–S3). Rarefaction analyses indicated that sequence coverage was sufficient (Supporting Information Fig. S1). Family‐level classification success using the manually curated DictDb 3.0 database (Mikaelyan *et al.,* 2015b) was 74% and 84% for sterile and reproductive castes respectively (Supporting Information Tables S2 and S3). Mothur analyses (Schloss *et al.,* 2009) produced 9314 OTUs across worker and soldier guts from 33 phyla, dominated by taxa previously reported to be abundant in fungus‐growing termites (Dietrich *et al.,* 2014; Mikaelyan *et al.,* 2015a; Otani *et al.,* 2014, 2016), and additional dada2 classification (Callahan *et al.,* 2016) was largely consistent with the mothur analyses (Supporting Information Tables S8 and S9). However, we nevertheless performed alpha‐ and beta‐diversity analyses using both approaches w support our main conclusions.

### *Compositional and functional differences across sterile caste gut communities*

**Figure 1:**
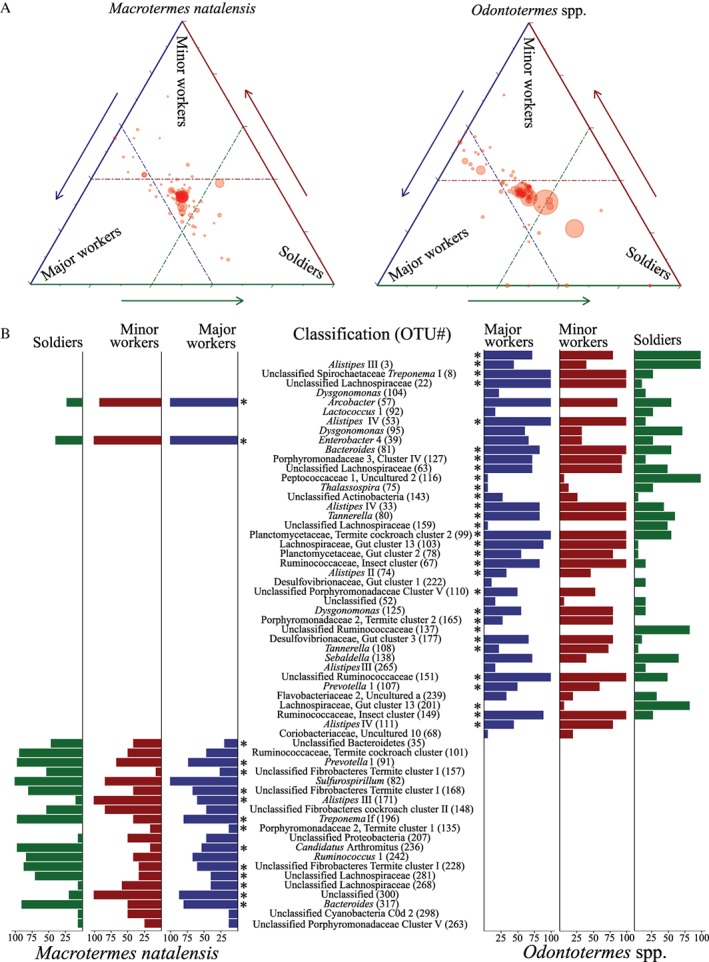
Distribution and differential abundance of gut bacteria across sterile castes. A. Ternary plot of the most abundant bacteria (> 70% relative abundances across gut communities) and their distribution between sterile castes, based on amplicon sequencing of the 16S rRNA gene (see Supporting Information for details). Each circle represents an OTU, and its size represents the relative abundance. The position of a circle represents the bacterium contribution to differentiating caste microbiota compositions, where the dashed lines represent 40% of the dissimilarity explained in the ordination analyses (based on the PCoA loading values). B. The proportional distributions of bacterial OTUs within a caste that explain > 40% of the microbial community variations between castes in a termite genus, with the scale representing the proportion of an OTU in a caste, that is, 100 means it is present in all individuals of a given caste. OTUs significantly different in at least one pairwise DESeq2 comparison are indicated with an asterisk (for the full results, see Supporting Information Table S12).

The vast majority of bacterial taxa were evenly distributed across minor workers, major workers and soldiers in *M. natalensis* and the two *Odontotermes* species (Fig. 1A). This included some of the most abundant bacterial OTUs and is likely driven by within‐colony trophallaxis, which transfers microbes from especially adults to newly hatched colony members (Hongoh, 2010; Nalepa, 2015). A subset of bacteria was differentially abundant across castes, evident from both ternary plots and subsequent DESeq2 (Love *et al.,* 2014) analyses, where the majority of the variable OTUs produced significant contrasts in at least one pairwise comparison (Fig. 1 and Supporting Information Table S12). We cannot rule out that more OTUs may be differentially abundant, as we chose a conservative cut‐off of 70% cumulative abundance which potentially selects against rare OTUs. The significant differences were almost exclusively driven by worker versus soldier comparisons, and only rarely driven by differences between minor and major workers (Fig. 1 and Supporting Information Table S12). *Arcobacter* and *Enterobacter* 4 were the only two OTUs that were common in workers of both termite genera (Fig. 1B), consistent with the DESeq2 results that the vast majority of the differentially abundant OTUs were distinct between *M. natalensis* and *Odontotermes* spp. (Fig. 1B and Supporting Information Table S12). This did not change in any appreciable way in the amplicon sequence variant (ASV) output from dada2 (Supporting Information Table S12). A phylogenetic analysis of the OTUs revealed that although they were not identical between termite genera, many were sister taxa, suggesting that they likely diversified from common ancestors (Supporting Information Fig. S2).

**Figure 2:**
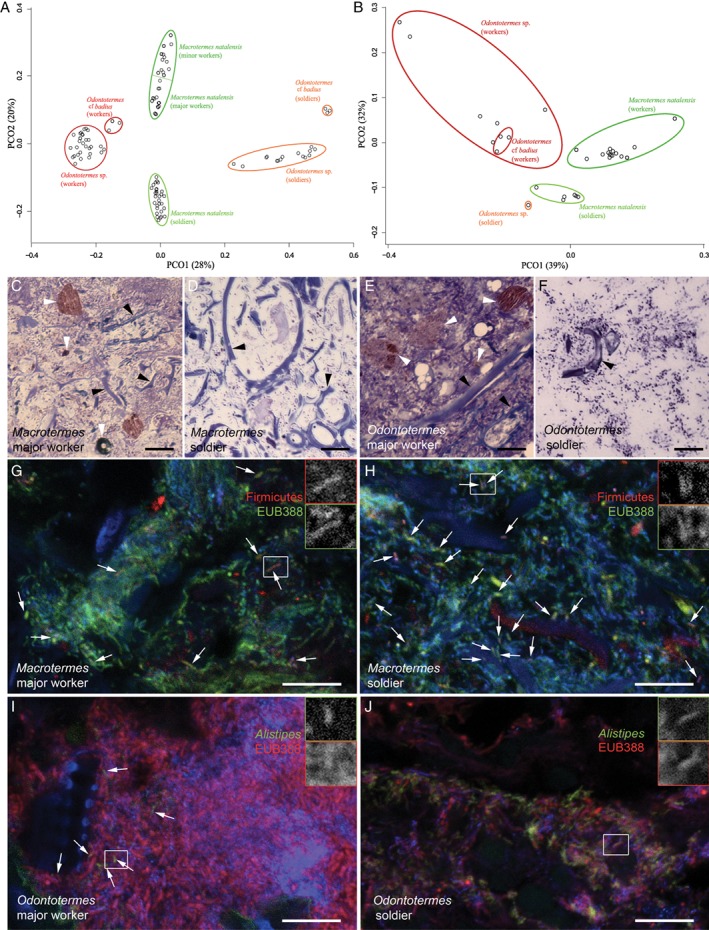
Structural and functional differences between gut communities of sterile castes. A. Bacterial community similarity analysis of the 108 sterile caste gut microbiotas, visualized using principal coordinate analysis (PCoA) based on Bray–Curtis distances. *p* values of Bray–Curtis distances between the eclipses were tested using an AMOVA test (*p* < 0.001, Supporting Information Table S6). B. PCoA of AZCL enzyme activities in different castes and ages. C–F. Different substrates in paunches (P3 region of the hindgut) of major workers and soldiers of *Macrotermes natalensis* (C, D) and *Odontotermes* cf. *badius* (E, F). Fungal material marked with black arrowheads, other types of substrate indicated by white arrowheads. Light microscopy of semi‐thin sections stained with toluidine blue. G, H. representative images of fluorescent *in situ* hybridisation with *Firmicutes*‐specific probe (red, arrows) in sections of the paunch (P3 of the hindgut) of a *Macrotermes natalensis* major worker (G) and soldier (H). All bacteria were stained with the domain‐specific probe EUB388 (green), DNA was stained with DAPI (blue). I, J. *Alistipes* III (green, arrows in I) in sections of the paunch (P3 of the hindgut) of an *O*. cf. *badius* major worker (I) and soldier (J), all bacteria were stained red (EUB388 probe), DNA was stained with DAPI (blue). *Alistipes* III was abundant in soldiers of *O*. cf. *badius* (J). The inserts show overlapping staining with two probes of target bacteria at higher magnification. Scale bars are 10 μm.

Differences between sterile castes were substantial enough to allow for distinct and significant separation in ordination plots (Fig. 2A; AMOVA *p* < 0.001; Supporting Information Table S6). The most distinct separations within sterile castes were between workers and soldiers (Fig. 2A and Supporting Information Table S12). The separate analyses did not allow for a comparison of reproductives versus sterile castes, but they were consistent with the combined analyses showing that caste and termite genus contributed the most to the observed differences in the merged (reproductives *and* sterile castes) dataset, driven mainly by species turnover (i.e. replacement; Supporting Information Table S11). This is consistent with recent work showing that the evolutionary histories of termite gut microbes are shaped by mixed‐mode transmission and acquisitions from environmental sources (Bourguignon *et al.,* 2018). The similarities within castes persist across geographical locations of colonies spanning more than 350 km (Fig. 2A), consistent with previous studies (Otani *et al.,* 2014, 2016), while differences between gut communities are less structured by colony of origin (Hongoh *et al.,* 2006; Otani *et al.,* 2016). The significant contrasts for beta diversity did not change in any appreciable way when we used the merged dataset from the dada2 analysis (Supporting Information Table S11).

To break down a complex substrate like lignocellulose, one would expect to find a highly diverse microbiota, which we do find in our study. This diversity is also reflected in the variety of glycosyl hydrolases that can act complementarily to utilize the individual components of lignocellulose (Liu *et al.,* 2013; Poulsen *et al.,* 2014). However, the complexity extends beyond polysaccharide degradation, as downstream products are utilized as substrates by different groups of bacteria along the degradation chain. Consistent differentially occurring bacteria among castes, thus, suggests that OTUs are likely to serve disparate functions in this process. Consistent with this assertion, *Alistipes* IV was more abundant in *Odontotermes* spp. workers than soldiers (Fig. 1B), and carbohydrate‐active enzyme analysis of *M. natalensis* guts suggest that *Alistipes* lineages have the enzymatic capacity to hydrolyse chitin and other fungal cell wall components (Liu *et al.,* 2013; Poulsen *et al.,* 2014). Lachnospiraceae (Firmicutes) are believed to be involved in non‐cellulosic saccharide degradation in fungus‐growing termites (Li *et al.,* 2016), consistent with a number of Lachnospiraceae OTUs being more abundant in soldiers (Fig. 1B and Supporting Information Table S2). This might suggest that workers transfer processed substrates (e.g. oligosaccharides) to soldiers via trophallaxis, and that these products are converted to fermentation products like butyrate and succinate by members of the Lachnospiraceae (cf., Li *et al.,* 2016). *Treponema*, a metabolically versatile genus involved in lignocellulose breakdown in other termites (Droge *et al.,* 2008; Graber and Breznak, 2004; Mikaelyan *et al.,* 2014) is generally scarce in fungus‐growing termites (Rahman *et al.,* 2015; Dietrich *et al.,* 2014; Otani *et al.,* 2014), but was nevertheless differentially distributed between sterile castes in a puzzling way: it was more abundant in *M. natalensis* soldiers than workers, while the opposite pattern emerged in *Odontotermes* spp. (Fig. 1B and Supporting Information Table S12).

Testing gut extractions on azurine‐crosslinked (AZCL) substrates showed that worker and soldier guts from *M. natalensis* and *Odontotermes* exhibited significant differences in their ability to degrade 14 different substrates (Fig. 2B and Supporting Information Table S4). Activities were highest in *M. natalensis* workers compared to their generic soldier and *Odontotermes* sterile castes (Fig. 2B, PERMANOVA *p* < 0.03) (Supporting Information Table S4). While *Odontotermes* worker guts were significantly lower in overall enzymatic capacities (Fig. 2B, PERMANOVA *p* < 0.02; Supporting Information Table S4), soldiers did not show any significant differences compared to other castes in this termite genus (Fig. 2B, PERMANOVA, *p* > 0.6; Supporting Information Table S4). In both termite genera, workers showed a greater capacity to degrade a wider range of substrates and to degrade plant‐derived substrates, for example, HE‐cellulose, xyloglucan, xylan and arabinoxylan, than soldiers (Supporting Information Table S4). This is consistent with a more diverse diet of workers and lends support for associations between gut microbial functions and compositions. Although we did document more diverse enzyme profiles in worker guts than in soldiers (Fig. 2), the resolution of this assay precludes explicit verification of patterns to the level of bacteria groups, where more targeted approaches and higher‐resolution assays will be needed.

### *Gut content characterization of termite sterile castes and* in situ *detection of caste‐specific bacteria*

Light microscopy revealed differences between worker and soldier gut substrates in the paunch (P3 region of the hindgut), with fungal substrates dominating soldier guts and worker guts containing a mixture of soil particles, fungus and plant material (Fig. 2C–F). These findings support the previously hypothesised predominant fungal diet of soldiers and more diverse worker diets (Hongoh *et al.,* 2006; Leuthold *et al.,* 2004; Eggleton, 2011). Albeit not quantitative in nature, we obtained further support for this distinction through confocal microscopy and FISH using probes specific for four bacterial OTUs in *Odontotermes* and one in *M. natalensis* (Lachnospiraceae, Bacteroidetes*, Alistipes* III and members of the Spirochaetaceae, including *Treponema*; Supporting Information Tables S7 and S12). The Lachnospiraceae OTU133 (Firmicutes) was more abundant and appeared more uniformly distributed in *M. natalensis* soldiers than workers (Fig. 2G and H), consistent with the sequencing results (Fig. 1B and Supporting Information Tables S2 and S12). This corroborates previous findings that Lachnospiraceae are more abundant in *O. formosanus* younger termites, feeding mainly on fungal nodules or degrading non‐cellulosic plant oligosaccharides (Li *et al.,* 2016). Similarly, *Alistipes* III (OTU8, Fig. 1B and Supporting Information Table S2) was significantly more abundant in *Odontotermes* soldier guts compared to worker guts (Fig. 2I and J), consistent with the sequencing results (Fig. 1B and Supporting Information Table S12). The remaining OTUs showed similar patterns, with morphologically different Bacteroidetes members being abundant in *Odontotermes* worker but not soldier guts (Supporting Information Fig. S3 and Table S7). Thus, even though the Bacteroidetes phylum is abundant in all sterile caste guts, dietary variation between castes likely causes representative OTUs within the phylum to be differently abundant. The probe targeting Spirochaetaceae was also more abundant in *Odontotermes* workers than soldiers (Supporting Information Fig. S3 and Table S7), further supporting the presence of caste‐enriched bacterial communities. No bacterial signal was detected with the negative probes (Supporting Information Fig. S4).

### *Queen and king gut microbiotas differ markedly from those of sterile termites*

**Figure 3:**
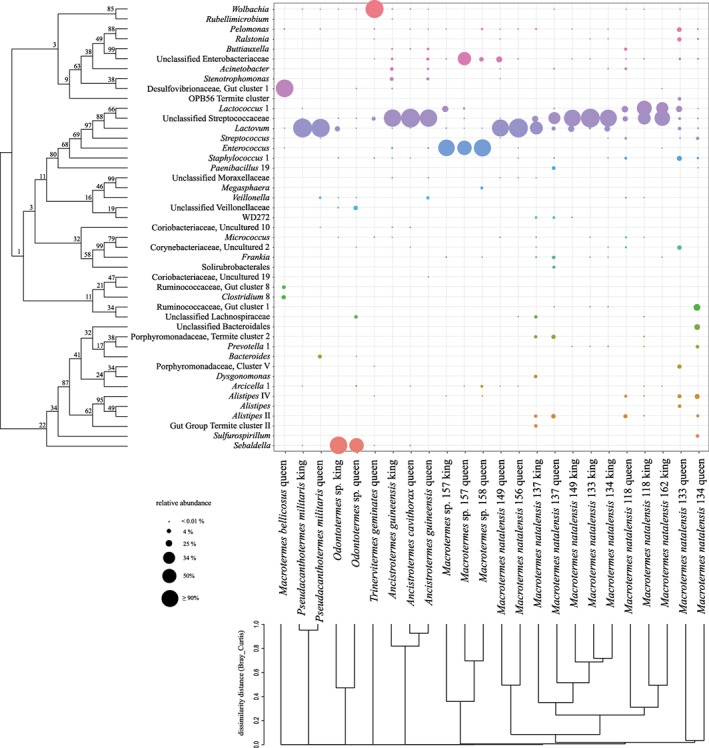
Gut bacterial communities in royal pairs from seven fungus‐growing termite species and a grass‐feeding *Trinervitermes geminatus* queen.The most abundant bacterial taxa in royal pair guts, accounting for > 0.1% relative abundance in each community. Circle sizes indicate the relative abundance of a bacterium in the corresponding gut, and colours represent different bacterial OTUs, which totals > 94% of all OTUs detected. *Wolbachia* dominated the grass‐feeding *T. geminatus* queen gut only (Supporting Information Table S3) driving this queen to be compositionally distinct from fungus‐growing termite queen and king gut microbiotas (Supporting Information Fig. S5). The ML tree at the left represents the phylogenetic relationships between the bacteria and the dendrogram at the bottom visualizes gut microbiota similarity analysis based on Bray–Curtis distances based on the most abundant bacterial taxa (dissimilarity analysis of the entire royal pair communities is presented in a PCoA plot in Supporting Information Fig. S5).

Previous compositional analyses of a single *M. natalensis* queen indicated that her gut was dominated by a single *Bacillus*‐like OTU (Poulsen *et al.,* 2014). We found that 1–3 bacterial genus‐level lineages dominate gut communities in each of 23 fungus‐growing termite royal pairs (13 queens and 10 kings) and a queen from the grass‐feeding termite species *T. geminatus*, with 10 bacterial lineages accounting for > 80% of all royal pair sequences (Fig. 3 and Supporting Information Table S3). Lactobacillales dominated *M. natalensis*, *Macrotermes* sp., two *Ancistrotermes* species and *P. militaris*, while the Desulfovibrionaceae gut cluster 1 OTU was the most abundant in a *M. bellicosus* queen gut and *Sebaldella* dominated an *Odontotermes* sp. queen and king (Fig. 3 and Supporting Information Table S3). Intriguingly, the dominant bacterial lineages in reproductives were absent or only present in low abundances in sterile caste guts (Supporting Information Tables S2 and S3), while OTUs that generally dominate sterile guts were absent or present in trace amounts only in royal pairs. The only exceptions to this was in queens of *M. natalensis* Mn118, Mn133 and Mn134 with more diverse and uniform bacterial communities (Fig. 3). With few bacterial genera dominating queen and king guts, alpha‐diversity of royal pair bacterial communities was significantly lower than in workers and soldiers (Supporting Information Fig. S6 and Table S5). Neither sampling site nor termite genus affected Shannon or evenness indices, while caste was highly significant for both our mothur or dada2 classification approaches (Supporting Information Table S5), consistent with the highly significant contrasts in the comparison of beta‐diversity between sterile and reproductive castes (Supporting Information Table S11). There was also a strong association between community structure and termite host species, indicating that conspecific royal pairs more commonly associate with the same or closely related bacteria (Fig. 3 and Supporting Information Fig. S5 and Table S6).

The presence of what appears to be specific and highly reduced diversity royal pair microbiotas suggest that the strict fungal diet and reproduction‐centred protected lifestyle, in which queens and kings are encapsulated in the royal chamber for the duration of their lifetime, is accompanied by simplified gut microbiotas. This resembles reported differences between workers and queens in the honeybee, where queens exhibit reduced microbial diversity and shifts in dominant gut bacteria (Kapheim *et al.,* 2015; Kwong and Moran, 2016; Tarpy *et al.,* 2015). Although the dominant bacteria differ between honey bee and fungus‐growing termite queens, it is intriguing that the termite royal pair guts are dominated by members of the Lactobacillales, which are believed to be involved in sugar uptake and fermentation in honeybee worker guts, and some lineages have antimicrobial properties (Ellegaard *et al.,* 2015; Killer *et al.,* 2014; Kwong *et al.,* 2014; Kwong and Moran, 2016). It is conceivable that termite royal pair gut microbes play similar roles but work to elucidate this is needed.

## Conclusions

Our survey of caste‐specific microbiotas in fungus‐farming termites confirm and expand previous work showing highly similar gut communities across colonies of the same termite species and clear distinctions between castes. Despite the continued exchange of symbionts via trophallaxis, a subset of bacteria proliferates differentially between soldiers and workers to likely serve different functions associated with differences in caste roles and diets. The reduced‐diversity queen and king gut microbiotas underline the influence of division of labour to the level of gut symbioses. While we provide preliminary evidence that worker and soldier guts are enzymatically distinct, the roles of the reduced assemblies of depauperate royal pair gut communities remain to be determined. Our findings, thus, set the stage for functional studies that can elucidate the relative importance of bacterial genera in termite castes and the mechanisms shaping gut compositions, particularly in the royal pair, who is expected to initiate their colony with microbial inocula for the first worker cohort. This implies, that they may carry more diverse gut microbiota at the onset of colony foundation but experience a shift in gut associations over the course of their extended lifespan.

## Data deposition

Clean reads are submitted to GenBank, where sample sequences with their accession numbers are under a single SRA submission with the accession SRP144287.

## Conflict of interest

All authors declare no conflicts of interests.

## Supporting information


**Fig. S1.** A. Rarefaction curves for sterile castes.
B. Rarefaction curves for reproductive castes.
**Fig. S2**. Maximum Likelihood phylogeny of differentially abundant OTUs between sterile castes.
Differentially abundant OTUs between *Odontotermes* workers and soldiers highlighted in pink boxes, between *M. natalensis* workers and soldiers in blue boxes, and between workers and soldiers in both termite genera in grey boxes.
**Fig. S3.** Representative images of fluorescent *in situ* hybridization (FISH) of the remaining three probes (two bacterial taxa) not included in Fig. 2G–J.
A, B. FISH with two probes (CF319a, green and CF319b, red) targeting members of Bacteroidetes in sterile castes of *Odontotermes* cf. *badius*. Note that bacteria stained with CF319a appear larger in minor workers (arrowheads).
C, D. FISH with a probe targeting members of the family Spirochaetaceae in sterile castes of *Odontotermes* cf. *badius*. Scale bars are 10 μm.
**Fig. S4.** Negative controls of fluorescent *in situ* hybridization using nonEUB388 probe in *M. natalensis* guts (A–D) and *O. badius* guts (E–G). Note the absence of signal.
All images are combining three channels: blue (DNA), green (probe staining and autofluorescence), red (autofluorescence). Scale bars are 10 μm.
**Fig. S5**. Queen and king gut microbiota similarity analysis (Bray–Curtis) visualized by principal coordinate analysis (PCoA) in R (R core team, 2013).
The symbols indicate a royal pair (circle = queen, triangle = king) and colours represents different termite taxa (grey = *Ancistrotermes guineensis*, brown = *Ancistrotermes cavithorax,* green = *Pseudacanthotermes*, blue = *Odontotermes*, light pink = *Macrotermes natalensis*, dark pink = *Macrotermes bellicosus* and purple = *Macrotermes* sp.).
**Fig. S6**. Diversity and evenness indices in gut microbiota from sterile and reproductive castes.
A. Shannon diversity indices of sterile and reproductive caste gut communities calculated with an R‐implemented script in Mothur and visualized by a violin plot in R.
B. Evenness index of termite sterile and reproductive caste gut bacterial communities calculated with an R‐implemented script in Mothur and visualized by a violin plot in R. * *p* < 0.00001 (*p*‐values are from Mann–Whitney–Wilcoxon tests in R, Supporting Information Table S5).

**Table S2.** Relative abundances of full bacterial OTUs identified in each termite sterile caste gut sample with full taxonomical levels presented. Click on the (+) sign or the numbers on the left panel to expand the taxon column.

**Table S3.** Relative abundances of full bacterial OTUs identified in each termite reproductive caste gut sample with full taxonomical levels presented. Click on the (+) sign or the numbers on the left panel to expand the taxon column.

**Table S1.** Termite colonies, sampling sites, the number of high‐quality 16S rRNA gene sequences obtained from MiSeq amplicon sequencing of the V3‐V4 region, and the number of OTUs.
**Table S4.** Termite colonies, sampling sites, sampling year and castes 155 and ages of termites used for the AZCL experiment, as well as the results of the measurements with one‐way PERMANOVA statistical analysis between soldier and worker from different species.
**Table S5.** Diversity index values of termite sterile and reproductive caste gut microbiotas, with Mann–Whitney–Wilcoxon statistical analyses of diversity indices between sterile and reproductive castes. Mn: *Macrotermes natalensis*, Od: *Odontotermes* sp., Od.b: *Odontotermes* cf. *badius*, A: *Ancistrotermes*, P: *Pseudacanthotermes*, S: soldier, JS: major soldier, NS: minor soldier, JW: major worker, NW: minor worker, Q: queen and K: king.
**Table S6.** Statistical analyses of Bray–Curtis dissimilarity distances between termite sterile castes, and reproductive caste gut microbiota structures, with analysis of molecular variance (AMOVA) was performed between gut microbiotas using R scripts implemented in mothur between distantly clustered microbial: between the different sterile caste guts from different termite species, and between different royal pair guts from different termite species, also between queen and king guts.
**Table S7.** FISH probes used for hybridisations and confocal microscopy.
**Table S8.** The results of dada2 analyses of royal pair guts, averaged across three technical replicates (amplifications and sequencing of the same sample). Results are given as relative abundances within samples. The top list provides a comparison of the results to those from the Mothur OTU analyses, with yellow highlights of samples for which the two analyses did not provide the exact same genus‐level classification.
**Table S9.** The results of dada2 analyses of sterile caste guts, averaged across three technical replicate amplifications and community sequencing. Results are given as relative abundances within samples and labels are: Mn: Macrotermes natalensis, Od: *Odontotermes* sp., Od.b: *Odontotermes* cf. *badius*, S: soldier, JS: major soldier, NS: minor soldier, JW: major worker, NW: minor worker.
**Table S10.** Results of linear mixed models, used to assess the main factors that affected OTU Shannon diversity and evenness. Two models were run using the R package vegan, with the colony used as a random effect and the Shannon or evenness indices as response variables. Main effects included in the models were the geographical location (site), t 187 he caste and the phylogeny. For the multiple comparisons we performed Tukey post‐hoc tests using the lsmeans package in R. Models were constructed using the alpha‐diversity indices calculated using the OTU tables from mothur (A), from dada2 (B) or a separate merged analysis using mothur (C).
**Table S11.** Results of beta‐diversity analysis. For the analyses we used Bray–Curtis distances calculated using the OTU table from the merged mothur analysis of all samples. The OTU table was rarefied at 13 000 reads. We used the betapart package in R which calculates the overall beta diversity but also separates beta‐diversity in turnover and nestedness. The former refers to beta diversity attributable to species replacement, whereas the latter indicates species loss or gain, that is, richness differences between the samples. We used as main effects, similar to before, the geographical location (site), the caste and the phylogeny, which were evaluated with ANOVA tests. For multiple comparison tests we used Tukey post‐hoc tests.
**Table S12.** The results of pairwise DESEQ2 comparisons of OTU abundances between castes in *Macrotermes natalensis* and *Odontotermes* spp. Only OTUs with significant differences are given.
