# Supplementary material for: Gut microbial compositions mirror caste‐specific diets in a major lineage of social insects
Source: Environ Microbiol Rep. 2019 Jan 8;11(2):196–205. doi: 10.1111/1758-2229.12728 (PMC6850719; doi:10.1111/1758-2229.12728)

Supplementary Figure S1. A) Rarefaction curves for sterile castes. B) Rarefaction curves for reproductive castes.

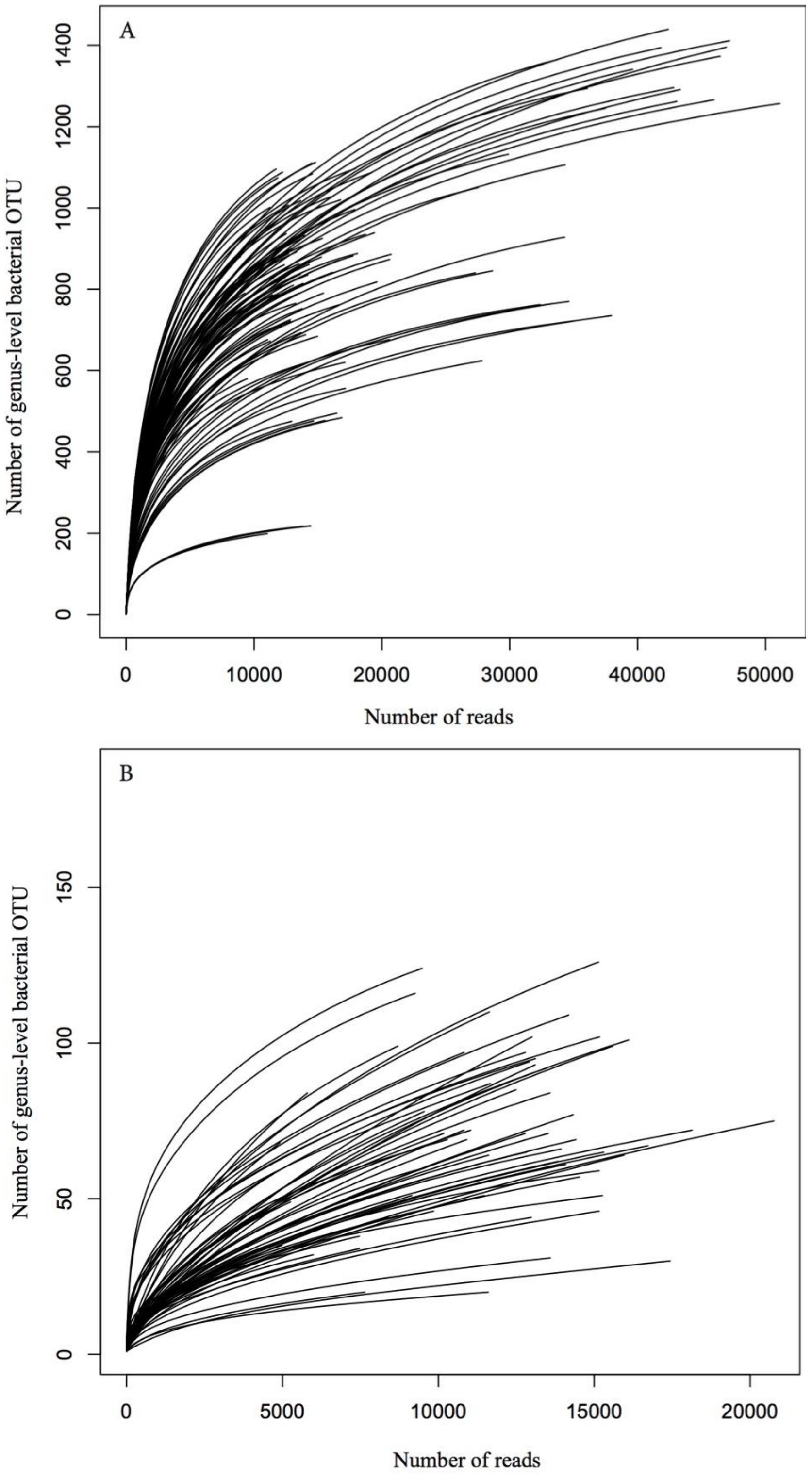

Supplementary Figure S2. Maximum Likelihood phylogeny of differentially abundant OTUs between sterile castes. Differentially abundant OTUs between *Odontotermes* workers and soldiers highlighted in pink boxes, between *M. natalensis* workers and soldiers in blue boxes, and between workers and soldiers in both termite genera in grey boxes.

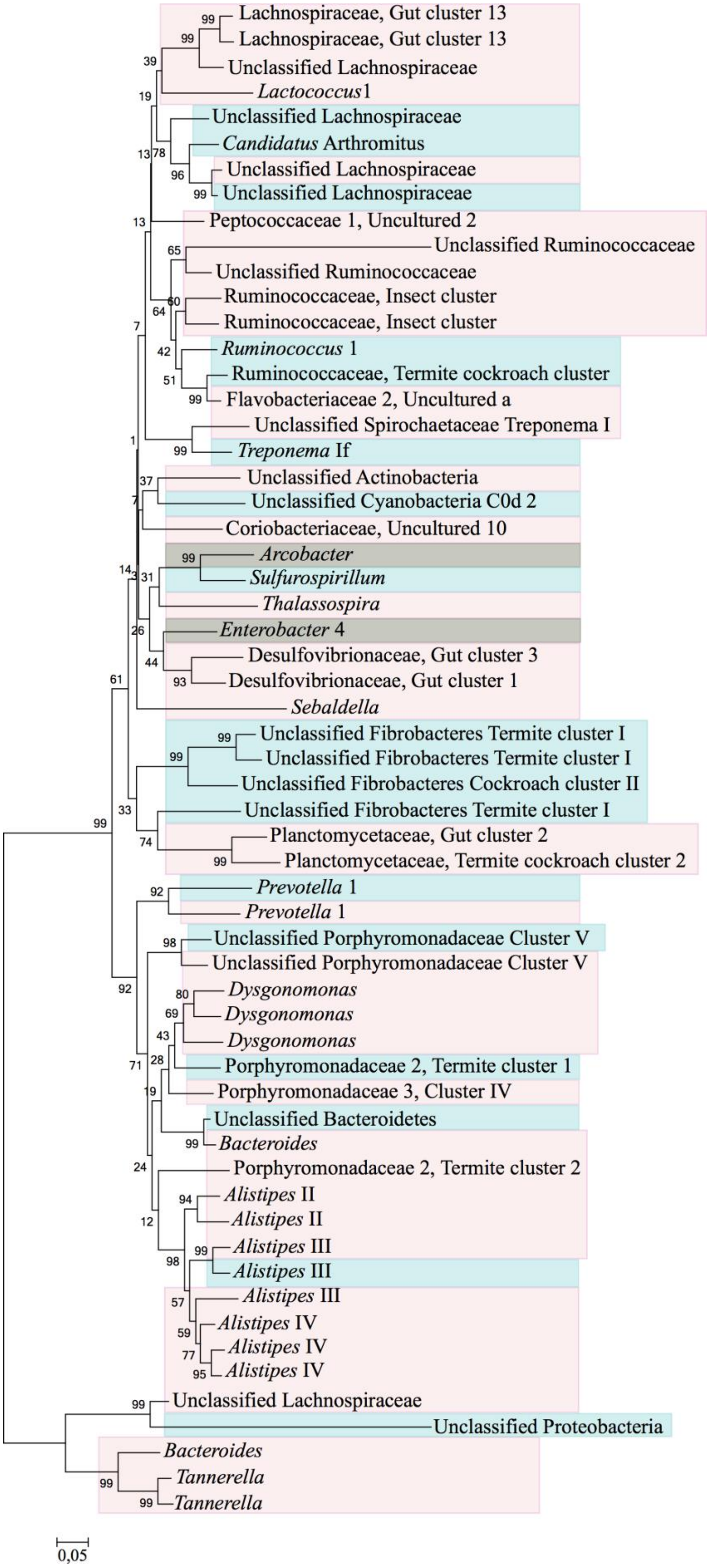

Supplementary Figure S3. Representative images of fluorescent *in situ* hybridization (FISH) of the remaining three probes (two bacterial taxa) not included in Figure 2G-J. A, B) FISH with two probes (CF319a, green and CF319b, red) targeting members of Bacteroidetes in sterile castes of *Odontotermes* cf. *badius*. Note that bacteria stained with CF319a appear larger in minor workers (arrowheads). C, D) FISH with a probe targeting members of the family Spirochaetaceae in sterile castes of *Odontotermes* cf. *badius*. Scale bars are 10 µm.

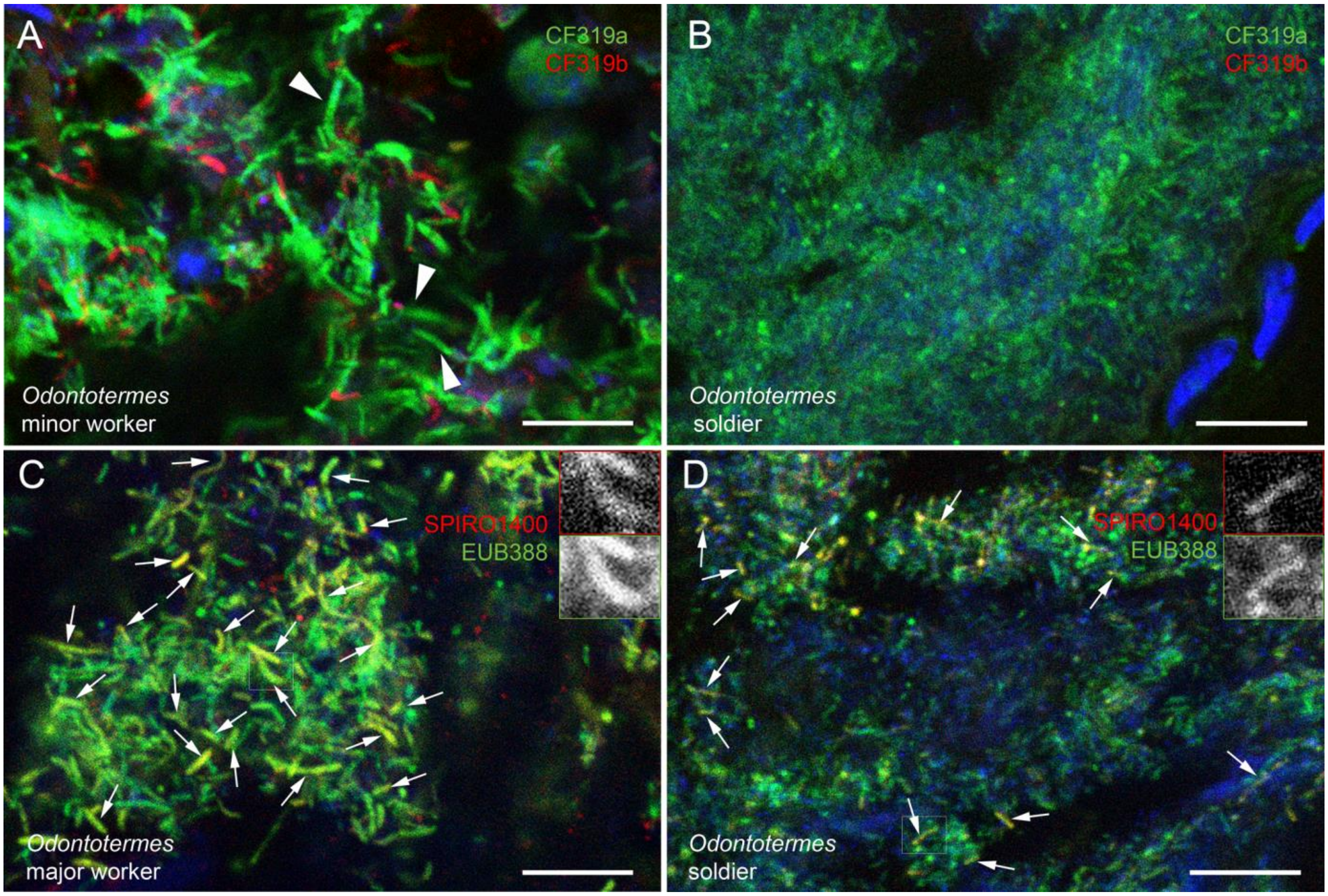

Supplementary Figure S4. Negative controls of fluorescent *in situ* hybridization using nonEUB388 probe in *M. natalensis* guts (A-D) and *O. badius* guts (E-G). Note the absence of signal. All images are combining three channels: blue (DNA), green (probe staining and autofluorescence), red (autofluorescence). Scale bars are 10 µm.

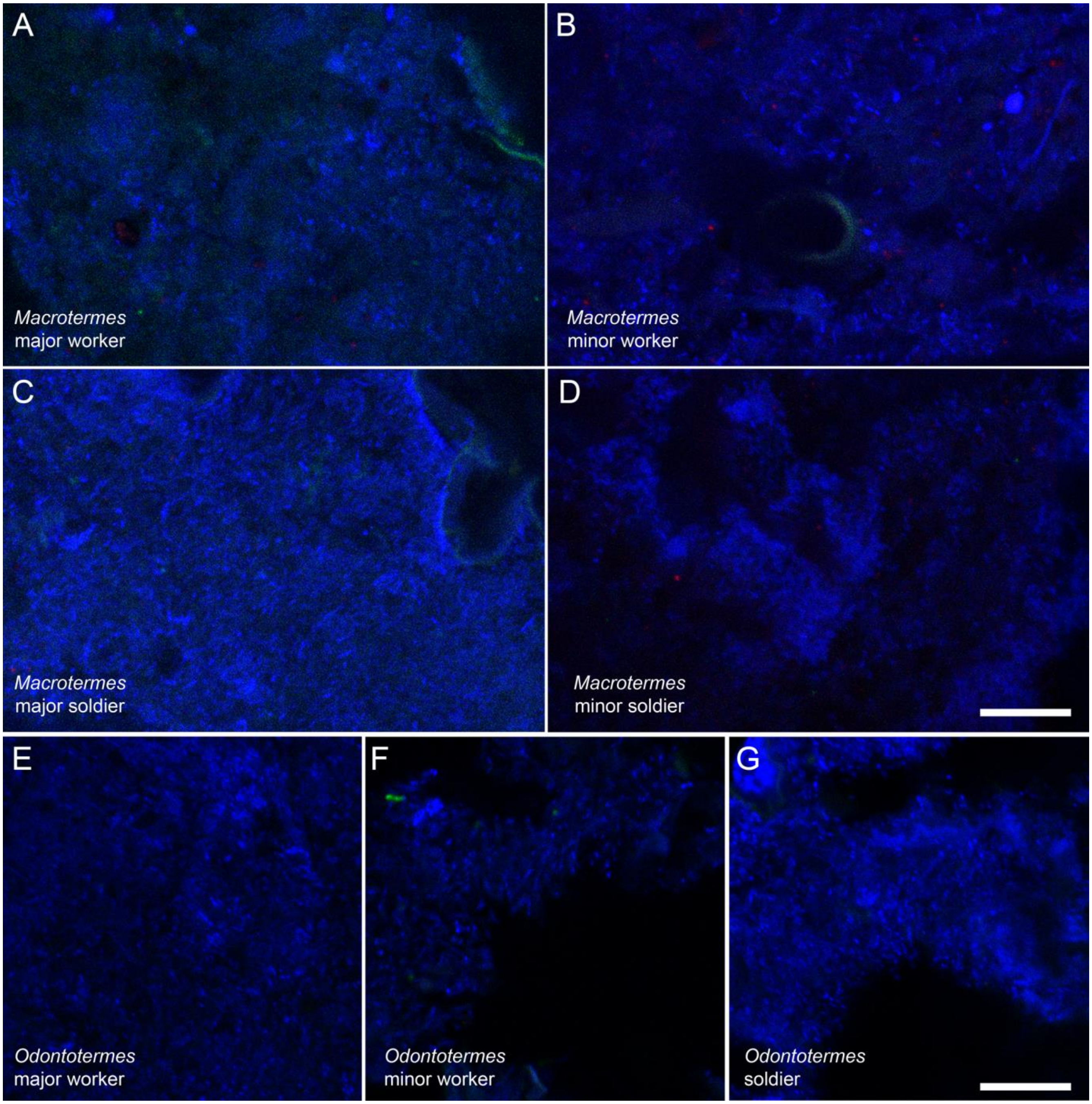

Supplementary Figure S5. Queen and king gut microbiota similarity analysis (Bray-Curtis) visualised by principal coordinate analysis (PCoA) in R (R core team, 2013). The symbols indicate a royal pair (circle=queen, triangle=king) and colours represents different termite taxa (grey=*Ancistrotermes guineensis*, brown=*Ancistrotermes cavithorax*, green=*Pseudacanthotermes*, blue=*Odontotermes*, light pink=*Macrotermes natalensis*, dark pink=*Macrotermes bellicosus* and purple=*Macrotermes* sp.).

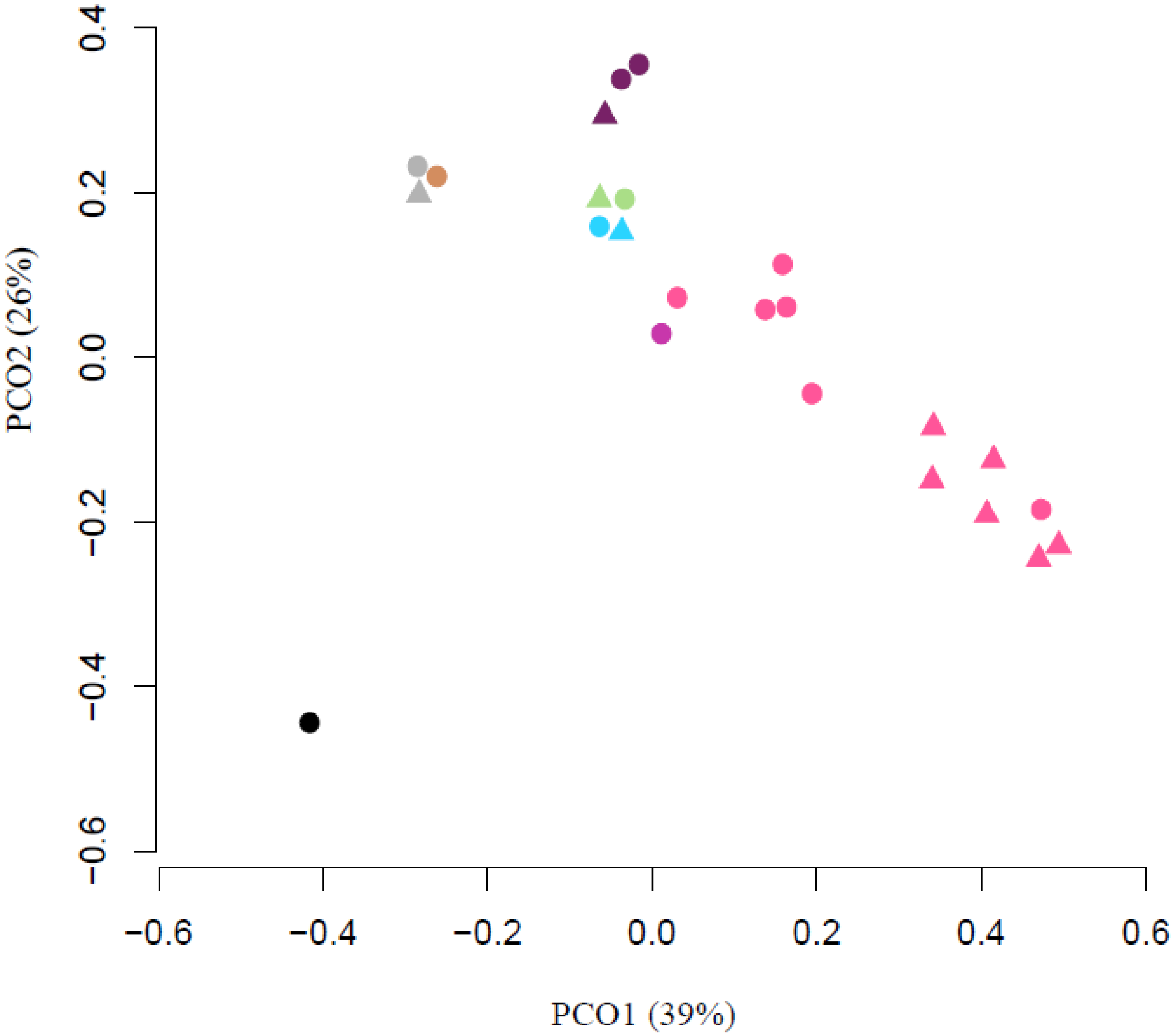

Supplementary Figure S6. Diversity and evenness indices in gut microbiota from sterile and reproductive castes. A) Shannon diversity indices of sterile and reproductive caste gut communities calculated with an R-implemented script in Mothur and visualised by a violin plot in R. B) Evenness index of termite sterile and reproductive caste gut bacterial communities calculated with an R-implemented script in Mothur and visualised by a violin plot in R. * $p < 0.00001$ (p-values are from Mann–Whitney–Wilcoxon tests in R, Table S5).

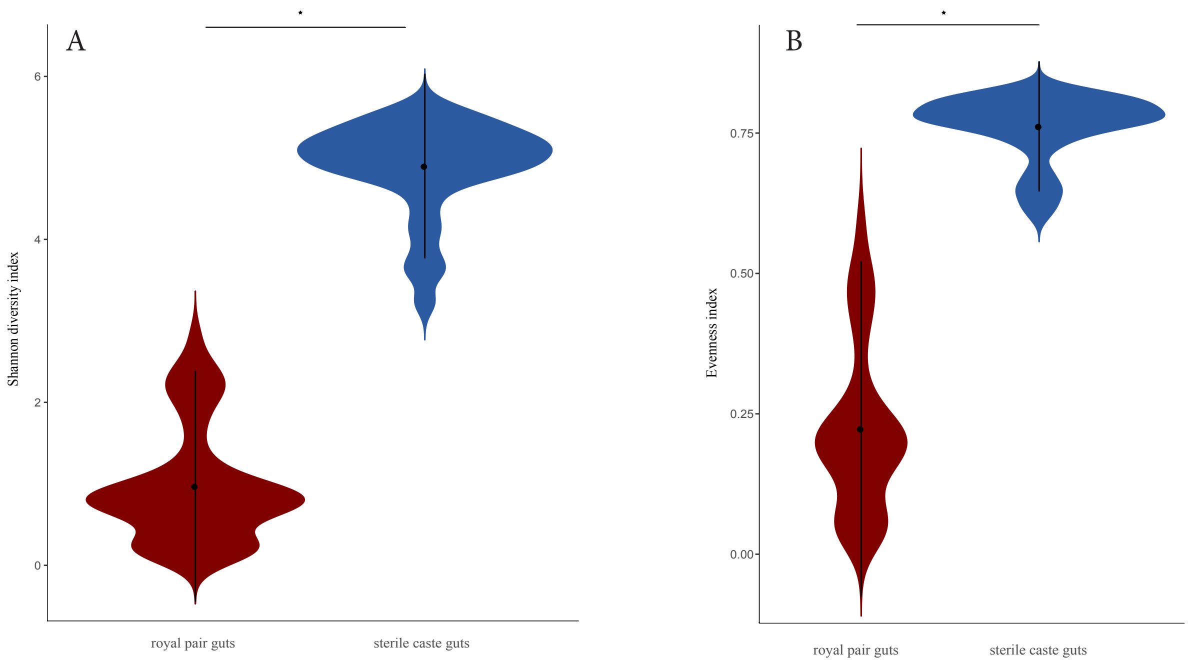

Supplement: Supplementary file 1 — Fig. S1. A. Rarefaction curves for sterile castes. B. Rarefaction curves for reproductive castes. Fig. S2. Maximum Likelihood phylogeny of differentially abundant OTUs between sterile castes. Differentially abundant OTUs between Odontotermes workers and soldiers highlighted in pink boxes, between M. natalensis workers and soldiers in blue boxes, and between workers and soldiers in both termite genera in grey boxes. Fig. S3. Representative images of fluorescent in situ hybridization (FISH) of the remaining three probes (two bacterial taxa) not included in Fig. 2G–J. A, B. FISH with two probes (CF319a, green and CF319b, red) targeting members of Bacteroidetes in sterile castes of Odontotermes cf. badius. Note that bacteria stained with CF319a appear larger in minor workers (arrowheads). C, D. FISH with a probe targeting members of the family Spirochaetaceae in sterile castes of Odontotermes cf. badius. Scale bars are 10 μm. Fig. S4. Negative controls of fluorescent in situ hybridization using nonEUB388 probe in M. natalensis guts (A–D) and O. badius guts (E–G). Note the absence of signal. All images are combining three channels: blue (DNA), green (probe staining and autofluorescence), red (autofluorescence). Scale bars are 10 μm. Fig. S5. Queen and king gut microbiota similarity analysis (Bray–Curtis) visualized by principal coordinate analysis (PCoA) in R (R core team, 2013). The symbols indicate a royal pair (circle = queen, triangle = king) and colours represents different termite taxa (grey = Ancistrotermes guineensis, brown = Ancistrotermes cavithorax, green = Pseudacanthotermes, blue = Odontotermes, light pink = Macrotermes natalensis, dark pink = Macrotermes bellicosus and purple = Macrotermes sp.). Fig. S6. Diversity and evenness indices in gut microbiota from sterile and reproductive castes. A. Shannon diversity indices of sterile and reproductive caste gut communities calculated with an R‐implemented script in Mothur and visualized by a violin plot in R. B. [file EMI4-11-196-s001.pdf]
